# Development and Clinical Evaluation of an Immunochromatography-Based Rapid Antigen Test (GenBody™ COVAG025) for COVID-19 Diagnosis

**DOI:** 10.3390/v13050796

**Published:** 2021-04-29

**Authors:** Doyeong Kim, Jihoo Lee, Jyotiranjan Bal, Seul Ki Seo, Chom-Kyu Chong, Jong Ho Lee, Hyun Park

**Affiliations:** 1 GenBody Inc., Cheonan 31116, Korea; dykim@genbody.co.kr (D.K.); jhlee@genbody.co.kr (J.L.); skseo@genbody.co.kr (S.K.S.); 2 Zoonosis Research Center, Department of Infection Biology, School of Medicine, Wonkwang University, Iksan 570749, Korea; jyoti_micro@yahoo.co.in; 3 Department of Laboratory Medicine, College of Medicine, Yeungnam University, Daegu 42415, Korea

**Keywords:** SARS-CoV-2, nucleocapsid, antigen detection test, RT-PCR, immunochromatography

## Abstract

Antigen tests for SARS-CoV-2 diagnosis are simpler and faster than their molecular counterparts. Clinical validation of such tests is a prerequisite before their field applications. We developed and clinically evaluated an immunochromatographic immunoassay, GenBody™ COVAG025, for the rapid detection of SARS-CoV-2 nucleocapsid (NP) antigen in two different clinical studies. Retrospectively, 130 residual nasopharyngeal swabs transferred in viral transport medium (VTM), pre-examined for COVID-19 through emergency use authorization (EUA)-approved real-time RT-PCR assay and tested with GenBody™ COVAG025, revealed a sensitivity and specificity of 90.00% (27/30; 95% CI: 73.47% to 97.89%) and 98.00% (98/100; 95% CI: 92.96% to 99.76%), respectively, fulfilling WHO guidelines. Subsequently, the prospective examination of 200 symptomatic and asymptomatic nasopharyngeal swabs, collected on site and tested with GenBody™ COVAG025 and EUA-approved real-time RT-PCR assay simultaneously, revealed a significantly higher sensitivity and specificity of 94.00% (94/100; 95% CI: 87.40% to 97.77%) and 100.00% (100/100; 95% CI: 96.38% to 100.00%), respectively. Clinical sensitivity and specificity were significantly high for samples with Ct values ≤ 30 as well as within 3 days of symptom onset, justifying its dependency on the viral load. Thus, it is assumed this can help with the accurate diagnosis and timely isolation and treatment of patients with COVID-19, contributing to better control of the global pandemic.

## 1. Introduction

The first case of the severe acute respiratory syndrome coronavirus 2 (SARS-CoV-2) infection was reported in December 2019 in Wuhan, China, [1,2]. The virus spread rapidly thereafter, resulting in a viral pandemic in 2020. According to the coronavirus disease 2019 (COVID-19) dashboard designed by the Center for Systems Science and Engineering at Johns Hopkins University, in December 2020, there were more than 72 million confirmed COVID-19 cases worldwide and more than 1.6 million registered deaths. These numbers are constantly rising. COVID-19 is mainly transmitted via droplets and aerosols [3], and, with the absence of vaccines, the only way to control the pandemic is to develop approaches for early and efficient diagnosis followed by patient isolation and treatment [4].

The causative agent of COVID-19 is SARS-CoV-2, a single-stranded, plus-sense RNA virus in the *Coronaviridae* family. The SARS-CoV-2 RNA genome encodes five major open reading frames that include non-structural replicase proteins as well as structural proteins [5]. Among them, the nucleocapsid (NP) gene is highly conserved and stable, with more than 90% amino acid homology with SARS-CoV and a low mutation rate [2,6,7]. As the NP is highly immunogenic, it is abundantly expressed in almost all coronavirus infections [8,9]. It is one of the early diagnostic markers of a SARS-CoV infection that can be detected up to one day prior to the onset of clinical symptoms [8]. Thus, the SARS-CoV-2 NP is a potential biomarker for the early diagnosis of COVID-19.

COVID-19 diagnosis mainly relies on the real-time reverse transcription-polymerase chain reaction (RT-PCR) assay, which is the current gold standard test for laboratory diagnosis of SARS-CoV-2 infections. However, RT-PCR is time-consuming and requires skilled personnel and costly equipment. Therefore, rapid and accurate tests for SARS-CoV-2 screening are essential to expedite diagnosis and prevent further transmission [10,11]. Antigen assays are immunoassays which detect specific viral antigens; thus, confirming a current viral infection. These tests, aimed at COVID-19 detection, are currently granted for emergency use authorization by the U.S. Food and Drug Administration as they are relatively inexpensive and can be used at the point of care. Clinical evaluation of the sensitivity and specificity of these tests is necessary for their field application. WHO-recommended interim guidelines specify a minimum of 80% sensitivity and 97% specificity for antigen-related diagnostic tests, compared with a molecular test, to be used for diagnosing COVID-19 patients. Expectedly, antigen tests are emerging as a promising candidate for early and rapid diagnosis, which may help prevent COVID-19 cases.

In this study, we developed and attempted a clinical evaluation of a rapid SARS-CoV-2 NP antigen detection test, GenBody™ COVID-19 Ag test (COVAG025), through its sensitivity and specificity towards COVID-19 diagnosis in two separate assessments. The performance of this immunochromatographic lateral flow assay for the detection of the SARS-CoV-2 NP antigen was compared with EUA-approved RT-PCR tests, retrospectively involving pre-confirmed residual nasopharyngeal swabs in VTM, as well as prospectively involving unknown symptomatic and asymptomatic individuals. The results were then further compared with EUA-approved RT-PCR tests. This clinical evaluation is essential for the implementation of the rapid antigen test for the screening of SARS-CoV-2-infected individuals, ensuring proper COVID-19 surveillance and patient management.

## 2. Materials and Methods

### 2.1. Ethics Statement

Two different studies were conducted according to International Standards of Good Clinical Practice. The retrospective clinical study was conducted at Yeungnam University Medical Center (YUMC), South Korea on 29 June 2020 and submitted to a properly constituted institutional review board (IRB), in agreement with local legal and ethical standards for formal approval of candidate diagnostic tests (IRB No.: YUMC 2020-06-058). The prospective clinical study was conducted at the Indian Council of Medical Research (ICMR)-approved Rao’s pathlab, India from 25 January 2021 to 3 February 2021 and submitted to a properly constituted institutional review board (IRB), in agreement with local legal and ethical standards for formal approval of candidate diagnostic tests (IRB No.: IRB00012217).

### 2.2. Preparation of Target Antibody

Codon-optimized SARS-CoV-2 nucleocapsid protein (NP) DNA, synthesized by Bioneer, South Korea [12], was cloned into *Escherichia coli* for the expression and production of recombinant NP [12] which was used for monoclonal antibody generation. Six-week-old BALB/c mice were injected subcutaneously with 50 μg of purified SARS-CoV-2 NP antigen in equal portions of complete Freund’s adjuvant (Sigma Aldrich, St. Louis, MO, USA) for initial immunization. Furthermore, three booster immunizations were administered at two-week intervals with a similar quantity of purified SARS-CoV-2 NP antigen in incomplete Freund’s adjuvant (Sigma, St. Louis, MO, USA). The mice received a final booster injection with 50 μg NP antigen intraperitoneally three days prior to cell fusion. 

The immunized mice were sacrificed, and isolated spleen cells were fused with the myeloma cell line SP2/0-Ag14 at a ratio of 5:1 using PEG 1500, as described by Kohler and Milstein [13]. The fused cells were then mixed with DMEM media supplemented with 20% (*v*/*v*) fetal bovine serum, 1% (*v*/*v*) HEPES, and 1% (*v*/*v*) HAT (Gibco; Grand Island, NY, USA) and cultured on 96-well plates (37 °C, and 5% CO2). Hybridoma culture supernatants were screened for high titer antibodies through indirect and novel antibody-capture ELISA, as described below.

### 2.3. Selection of Target MAb Pairs

#### 2.3.1. Indirect Enzyme-Linked Immunosorbent Assay (ELISA) to Confirm Selectivity

**Figure 1:**
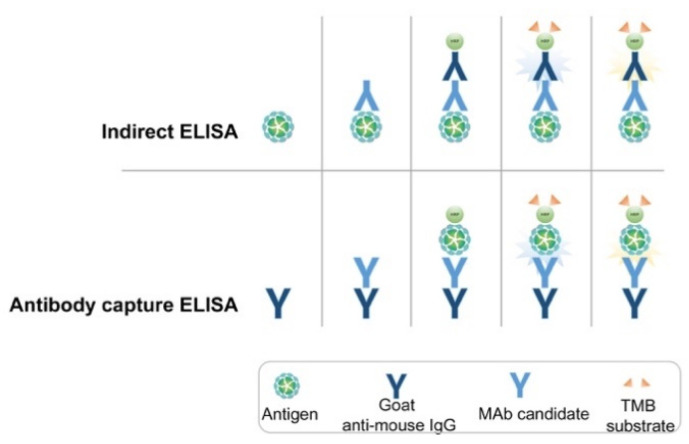
Schematic representation of indirect and antibody-capture ELISA assays used for screening and selection of MAb pairs.

Indirect ELISA assays (Figure 1) were used to confirm the selectivity of the MAbs against the purified recombinant NP of SARS-CoV-2. The purified NP (0.1 µg/well) was used to coat the wells of NUNC Maxisorp 96-well ELISA plates. The plates were washed three times with phosphate-buffered saline (PBS) + 0.05 % Tween 20 (PBST) and blocked with 0.5 % bovine serum albumin (BSA) for 2 h at 37 °C. Subsequently, 100 μL of hybridoma cell supernatants was added to the wells, which were then incubated for 30 min at 37 °C. The plates were washed with PBST three times and goat anti-mouse IgG-horseradish peroxidase (HRP)-conjugated secondary antibody (Sigma Aldrich, St. Louis, MO, USA) was added and incubated for 30 min at 37 °C, followed by a washing step. To detect the response, the substrate TMB (3,3′,5,5′-tetramethylbenzidine) solution (BioFX Laboratories Inc., Owings Mills, MD, USA) was added and the plates were incubated under similar conditions. The reaction was stopped with 0.5 N H_2_SO_4_ and absorbance was measured at 450 nm in a microplate reader (microplate spectrophotometer; Bio-Rad Laboratories Inc., Hercules, CA, USA).

#### 2.3.2. Antibody-Capture ELISA for Selection of Mab Pairs Against NP

In the novel antibody-capture ELISA assay (Figure 1), NUNC Maxisorp 96-well ELISA plates were coated with goat anti-mouse IgG (1 μg/mL) and blocked with 0.5% BSA. Next, 100 μL hybridoma cell supernatants were added to each well and the plates were incubated for 30 min at 37 °C. After washing, HRP-conjugated recombinant NP antigen was added, and the plates were incubated as before. The enzymatic reaction was visualized as described above. HRP-conjugated recombinant NP antigen was prepared according to the manufacturer’s instructions (Peroxidase Labeling Kit; Sigma Aldrich, St. Louis, MO, USA).

### 2.4. Assembly of Rapid Diagnosis Test (RDT) Kit

Several RDT kits have been designed and are awaiting clinical approval for use in COVID-19 diagnosis. With slight modifications from earlier studies [14,15,16], we assembled the GenBody™ COVID-19 Ag test as follows. The MAbs against SARS-CoV-2 NP (1 mg) were conjugated with previously prepared colloidal gold particles (100 mL) [12]. The MAb-gold conjugates were precipitated by centrifugation and dissolved with PBS containing 0.1% BSA to adjust the OD_450_ to 10. The conjugates were then treated on a glass fiber and dried to prepare the conjugator pads. The MAbs against SARS-CoV-2 NP were dispensed and immobilized at the appropriate positions on a nitrocellulose membrane (2.5 mg/mL). Goat anti-mouse IgG (1 mg/mL; Arista Biologicals Inc., Allentown, PA, USA) was dispensed and immobilized on the control line of the membrane. The buffer pad was prepared by treating cellulose paper (Grade 319; Ahlstrom Inc., Alpharetta, GA, USA) with 0.1 M carbonate (pH 9.0). The absorbance pad consisted of untreated cotton paper. All pads were partially overlapped to enable the migration of the sample and buffer solution along the strip.

### 2.5. Limit of Detection (LOD) of GenBody™ COVID-19 Ag Test

To determine the LOD of the developed kit, cultured and inactivated SARS-CoV-2 virus obtained from Zeptomatrix Inc. (Buffalo, NY, USA) and the recombinant SARS-CoV-2 NP from the previous study [12], along with anti-NP MAbs 3C3 and 2F4, were used. An IC Reader (GenBody Confiscope G20) was used to quantitate the intensities of the test lines. The G20 cut off value was set at 100,000 and samples with a G20 value > 100,000 were considered positive. The analytical sensitivity of the GenBody™ COVID-19 Ag test was evaluated by the comparison of TCID_50_ values/concentration of recombinant SARS-CoV-2 NP antigen with the corresponding G20 values. The lowest TCID_50_ value/concentration of recombinant NP antigen at which the G20 value was assessed to be positive was designated as the LOD of the GenBody™ COVID-19 Ag test.

### 2.6. Clinical Specimens

In the clinical evaluation study conducted at Yeungnam University Medical Centre, South Korea, tests were performed according to the instructions for use of the GenBody™ COVID-19 Ag test (COVAG025) with residual Nasopharyngeal swabs transferred in VTM, ESwap^TM^ 482C (COAPN Diagnostic Inc., Carlsbad, CA, USA)/T-SWAB TRANSPORT^TM^ CTM (Noble Biosciences Inc., Gyeonggi-do, Korea), from 30 positive and 100 negative specimens confirmed by the EUA-approved real-time RT-PCR assay Allplex™ 2019-nCoV (Seegene Inc., Seoul, Korea) 

**Figure 2:**
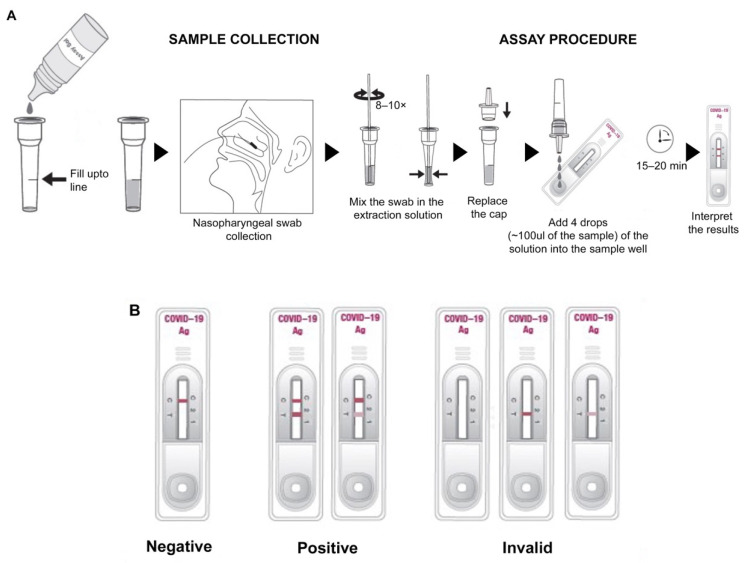
Schematic representation of (**A**) sample collection and assay procedure, (**B**) interpretation of test results of the RDT strip.

For a prospective clinical evaluation study at Rao’s pathlab, India, a second set of clinical samples, including nasopharyngeal swabs from symptomatic and asymptomatic individuals from multiple centers, were collected (Figure 2A) and transferred in VTM, followed by analysis within 24 h with the GenBody™ COVAG025 and compared simultaneously with the EUA-approved EURORealTime SARS-CoV-2.

In both the cases, sample collection was performed according to the specimen collection guidelines of the Centers for Disease Control and Prevention (CDC). For nasopharyngeal sample collection, a sterile swab was carefully inserted into the nostril that presented the most secretion upon visual inspection. It was gently rotated, pushing the swab further, until resistance was met at the level of the turbinate (less than one inch into the nostril), followed by repeated rotation against the nasal wall (Figure 2A). The nasopharyngeal swab was then swirled several times in the vial containing VTM/extraction solution. The extraction solution containing the specimen could be stored at room temperature for up to 1 h or at 2–8 °C (36–46 °F) for up to 12 h.

### 2.7. Clinical Evaluation of COVAG025

Ten microliters of specimen were loaded into the sample well of the device and three drops (~100 μL) of buffer solution were subsequently loaded into the sample well (Figure 2A). Results were interpreted within 15 min. The appearance of the control and test lines was assessed after 15 min. An IC Reader (GenBody^TM^ Confiscope G20, Chungcheongnam-do, Korea) was used to quantitate the intensities of the test lines. The G20 cut off value was set at 100,000 and samples with a G20 value > 100,000 were considered positive. Tests were considered valid if a color appeared at the control line (Figure 2B). If a red color appeared at the test line, the specimen was supposed to contain SARS-CoV-2 Ag.

## 3. Results

### 3.1. Generation, Screening and Selection of MAbs against SARS-CoV-2 NP

**Figure 3:**
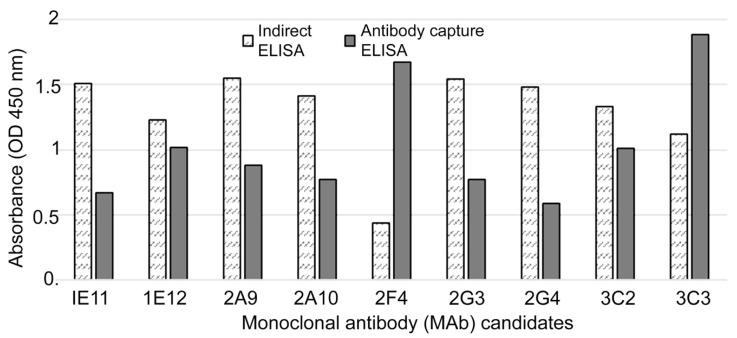
Comparison of indirect and antibody-capture ELISA for screening and selection of efficient MAbs.

Using recombinant SARS-CoV-2 NP as an immunogen, MAbs were generated. Nine hybridoma cell lines that stably secreted MAbs were identified based on their high absorbance values in the HRP-conjugated antigen-based antibody-capture ELISA screening. To screen the extremely sensitive and specific MAbs against SARS-CoV-2 NP, we applied two ELISA methods: the indirect ELISA and antibody-capture ELISA. The novel antibody-capture ELISA was found to be more reliable for the screening of hybridoma clones. Clone 2F4, which exhibited extremely low optical density, was easily identified using this screening method (Figure 3). MAb 2F4 showed an extremely low titer when the indirect ELISA was used for screening, whereas a high titer was observed with the newly developed antibody-capture ELISA.

**Figure 4:**
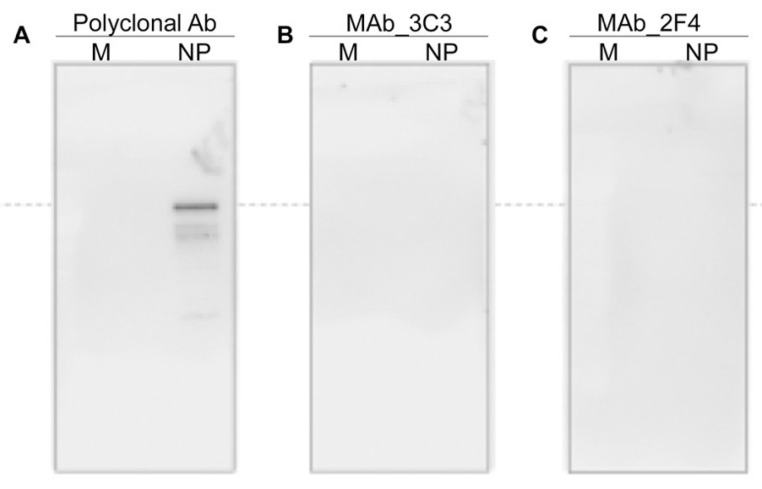
Western blot analysis to show the ineffectiveness of screening of MAbs through indirect ELISA. (**A**) Treated with polyclonal Ab against NP, (**B**,**C**) treated with MAbs.

Furthermore, MAbs 2F4 and 3C3 were non-reactive in Western blot using the membrane transferred after SDS-PAGE (Figure 4). This indicated that, despite high affinity, MAbs may have failed to bind to antigens when the traditional indirect ELISA method for hybridoma screening was used.

### 3.2. Selection of MAb Pairs for Use in GenBody™ COVID-19 Ag Test (COVAG025)

**Figure 5:**
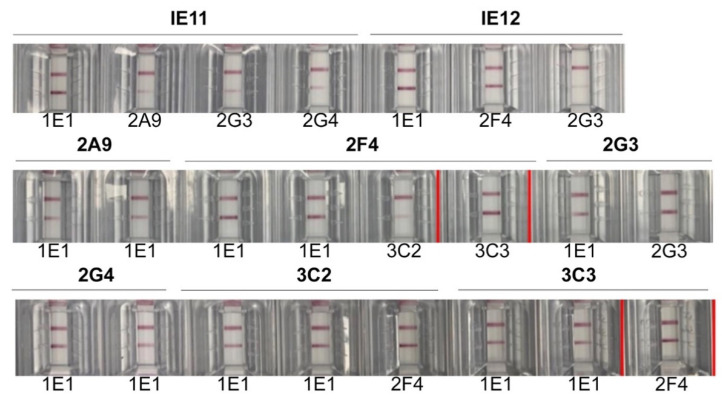
Selection of MAb pairs through sandwich LFA method. The effective MAb pair is marked with red-colored boundaries.

The purified MAbs generated against SARS-CoV-2 NP were further screened through a sandwich LFA assay to select the effective pair for the detection of recombinant SARS-CoV-2 NP (Figure 5). MAbs 3C3 and 2F4 exhibited the best sensitivity towards SARS-CoV-2 recombinant NP protein. Consequently, MAbs 3C3 and 2F4 were used as the capture antibody in the membrane and to conjugate gold composites, respectively in the GenBody™ COVID-19 Ag test strip.

### 3.3. Limit of Detection (LOD) of GenBody™ COVID-19 Ag Test (COVAG025)

**Figure 6:**
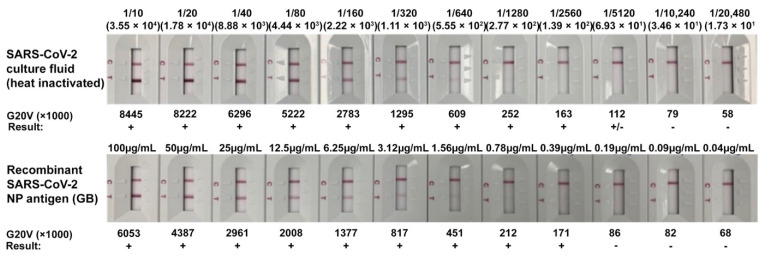
Limit of detection of GenBody™ COVID-19 Ag test using inactivated SARS-CoV-2 and recombinant SARS-CoV-2 NP.

The analytical sensitivity of the GenBody™ COVID-19 Ag test was evaluated following the procedures described in the materials and methods. The TCID_50_/_mL_ of the inactivated SARS-CoV-2 for the corresponding lowest G20 positive value was determined to be 6.93 × 10^1^ whereas, 0.39 μg/mL of the recombinant SARS-CoV-2 NP represented the lowest corresponding G20 positive value (Figure 6). Thus, the LOD of the GenBody™ COVID-19 Ag test (COVAG025) was determined to be 6.93 × 10^1^ TCID50/mL for inactivated SARS-CoV-2 and 0.39 μg/mL for recombinant SARS-CoV-2 NP.

### 3.4. Clinical Evaluation of GenBody™ COVID-19 Ag Test in Testing Pre-Confirmed COVID-19 Samples

Among a total of 130 residual nasopharyngeal swabs in VTM from individuals who either visited or were hospitalized at Yeungnam University Medical Centre, 30 were confirmed positive for COVID-19 and 100 were designated negative, based on the EUA-approved real-time RT-PCR assay Allplex™ 2019-nCoV (Seegene Inc., Seoul, Korea). The diagnostic accuracy results estimated the clinical sensitivity of the GenBody™ COVID-19 Ag test assay to be 90.00% (95% CI: 73.47–97.89%) with a positive predictive value of 93.10% (95% CI: 77.23–99.15%) and three false negatives, while the clinical specificity was estimated to be 98.00% (95% CI: 92.96–99.76%) with a negative predictive value of 97.03% (95% CI: 91.56–99.38%) and two false positives, as shown in Table 1. Based on this, the overall accuracy of the test was estimated to be 96.15%, (95% CI: 91.25–98.74%).

#### Association of Test Results of GenBody™ COVID-19 Ag Test with Viral Load

**Figure 7:**
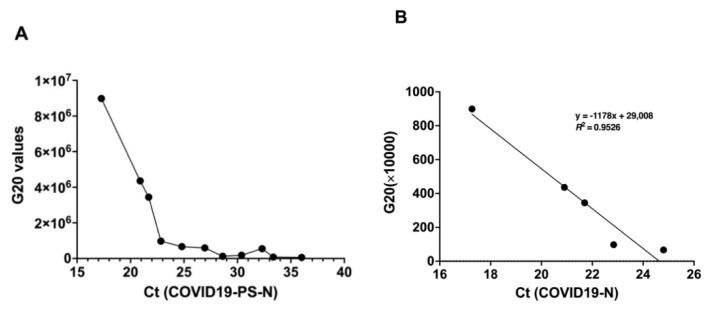
Correlation analysis of G20 values of RDT strip with the corresponding Ct values (SARS-CoV-2-N gene) of the samples. (**A**) Inter-dependance between G20 values and Ct values, (**B**) linear regression analysis.

Increasing Ct values indicated a decrease in the viral load. The clinical sensitivity of the GenBody™ COVID-19 Ag test was estimated to be 100% (95% CI: 78.20–100%) for samples with Ct values ≤ 25; this slightly decreased to 91.67% (95% CI: 61.52–99.79%) with Ct values between 25 and 30, as recorded through the RT-PCR assay Allplex™ 2019-nCoV for N gene (Table 2). Several constraints prevented the acquisition of more samples and, due to the extremely low number of samples with Ct values < 30, it was inappropriate to come to a conclusion about their sensitivities. Further confirmation of the dependance of the test results of the GenBody™ COVID-19 Ag test with the viral load was confirmed through a correlation analysis between the Confiscope G20 values of the RDT strip and the corresponding Ct values (Figure 7A,B). Before the Ct values for NP were < 25, the G20 values linearly correlated with the corresponding Ct values for NP with R^2^ equivalent to 0.953. Since the Confiscope readings represented a qualitative measurement of the band intensity, a further decrease in band intensity for Ct values above 25 could not establish a correlation between the G20 and Ct values > 25.

Furthermore, the sensitivity among samples collected within 3 days (Table 3) after disease onset was slightly higher (100%) than for samples collected ≥ 4 days of disease onset (89.47%). The above observations indicated a dependency of the clinical sensitivity of GenBody™ COVID-19 Ag test upon the viral load.

### 3.5. Prospective Clinical Evaluation of GenBody™ COVID-19 Ag Test in Testing Suspected COVID-19 Samples

Nasopharyngeal swabs from a total of 200 patients were tested at the point of collection site with proper consent for specimen collection and provision of information. Real-time PCR analysis with EUA-approved EURORealTime SARS-CoV-2 indicated 100 out of 200 samples were COVID-19-positive. Testing with the GenBody™ COVID-19 Ag test indicated an overall detection sensitivity and specificity of 94.00% (94/100; 95% CI: 87.40–97.77%) and 100.00% (100/100; 95% CI: 96.38–100.00%), respectively, with six false negatives and no false positives (Table 4). Thus, the negative predictive value was estimated to be 94.34% and the positive predictive value to be 100%. Based on this, the overall accuracy of the test was estimated to be 97.00%, (95% CI: 93.58–98.89%).

Furthermore, the clinical sensitivity of the GenBody™ COVID-19 Ag test in testing suspected samples was estimated to be 90.74% (95% CI: 79.70–96.92%) for samples with Ct values ≤ 25. This slightly increased to 97.78% (95% CI: 88.23–99.94%) with Ct values between 25 and 30, as recorded through the RT-PCR assay EURORealTime SARS-CoV-2 (Table 5). Since only one sample had a Ct value over 30, it was inappropriate to determine the sensitivity. Furthermore, sensitivity among samples collected within 3 days (Table 6) after disease onset was higher (95.52%) than for samples collected ≥ 4 days of disease onset (90.91%). This further confirmed the dependance of the clinical sensitivity of the GenBody™ COVID-19 Ag test upon the viral load.

## 4. Discussion

The containment of rapidly surging SARS-CoV-2 cases requires faster and accurate diagnostics. Limitations of the gold standard nucleic acid amplification tests (NAATs) [17] pave the way for quicker and accurate antigen/antibody-mediated rapid diagnostic tests (RDTs). The high demand for rapid and accurate diagnostics led to the production of a large number of RDTs, but most of them still await clinical evaluation. We developed a novel RDT, GenBody^TM^ COVID-19 Ag test COVAG025, with a LOD of 6.93 × 10^1^ for SARS-CoV-2 inactivated virus and 0.39 µg/mL for recombinant SARS-CoV-2 NP. We describe here the results of two diverse clinical evaluation studies of COVAG025 at two different locations in comparison to EUA-approved real-time RT-PCR assays following the norms and guidelines on the review and approval of in vitro diagnostic devices for COVID 19, as recommended by the South Korean Ministry of Food and Drug Safety. The overall diagnostic sensitivity/specificity of the retrospective and prospective studies was 90/98% and 94/100%, respectively. This was in compliance with WHO guidelines for the evaluation of Ag-RDTs, which recommends a minimum of 80% sensitivity and 97% specificity for Ag-RDTs. Our data was compliant with other independent clinical evaluations [18,19] with low rates of false positivity.

Antigen-based RDTs for SARS-CoV-2 are reported to target multiple antigens, including the SARS-CoV-2 spike or nucleocapsid protein. Recent studies support the efficacy of the nucleocapsid protein as a detection target in these types of antigen-based assays [8,20]. Reports involving SARS-related viruses demonstrate the secretion of high levels of the nucleocapsid protein relative to the other viral proteins [21]. Thus, the GenBody^TM^ COVID-19 Ag test COVAG025, utilizing NP as the target antigen, was assumed to be successful in detecting SARS-CoV-2.

One of the unique strengths of this study is the clinical evaluation of both retrospective residual samples as well as prospective unknown samples from the point of collection. The diagnostic sensitivity and specificity were both elevated in the diagnosis of unknown samples at the point of collection, rather than residual samples stored in VTM. The number of false positives also decreased in the diagnosis of unknown samples at the collection site, elevating the positive prediction value to 100%.

Ag-RDT has been reported to have high specificity, but its use in clinical diagnosis is sometimes questioned because of its low sensitivity. Recent studies showed Ag-RDT is unable to detect SARS-CoV-2 at a Ct value of more than 19 [22]. Other reports have revealed the diagnostic sensitivity of Ag-RDT to be 95% at Ct values < 25 but declines drastically to 20–40% when the Ct value is > 25 [23]. In contrast to all these observations, the GenBody^TM^ COVID-19 Ag test COVAG025 showed diagnostic sensitivities of 90–100% at Ct levels ≤ 25 in residual samples and unconfirmed fresh samples, which also further persisted at Ct levels > 25. This proved the superior clinical diagnostic ability of the GenBody^TM^ COVID-19 Ag test COVAG025 in diagnosing SARS-CoV-2.

In asymptomatic individuals, the overall sensitivity of the GenBody^TM^ COVID-19 Ag test was estimated to be 75% (95% CI: 19.41–99.37%). Despite not displaying any noticeable symptoms, these cases may contribute to the spread of the virus. This study’s results provide substantial evidence that the point-of-care GenBody^TM^ COVID-19 Ag test can accurately identify SARS-CoV-2 antigens in people with suspected COVID-19, as well as in asymptomatic people with a high viral load. Previous reports have shown that asymptomatic persons have similar viral loads to symptomatic persons [24,25]; therefore, this Ag-RDT could be used in this type of population for proper monitoring and isolation.

## 5. Conclusions

The clinical evaluation of the GenBody^TM^ COVID-19 Ag test COVAG025 for the rapid detection of SARS-CoV-2 antigen in 130 residual confirmed specimens and 200 nasopharyngeal samples from suspected patients from the site of collection, in comparison with results obtained by using other commercialized RT-PCR assays, exhibited comparable sensitivity and selectivity. Although the sample size was small due to the various constraints of sample collection, the results indicated a trend towards the effectiveness of the GenBody^TM^ COVID-19 Ag test COVAG025 in the diagnosis of COVID19 based on viral load. Given the speed, low complexity and accuracy of the GenBody^TM^ COVID-19 Ag test COVAG025, it is predicted to be suitable for the rapid identification of positive patients. Thus, we believe the GenBody™ COVID-19 Ag test has potential for use as a simple and rapid SARS-CoV-2 antigen detection test, especially in high prevalence areas.

## Figures and Tables

**Table 1:** Results of the test using the GenBody COVID-19 Ag test (COVAG025).

Evaluation Results of Test Equipment (GenBody COVID-19 Ag Test (COVAG025)	Confirmed Results through RT-PCR		Total
	Positive	Negative	
Positive	27	2	29
Negative	3	98	101
Total	30	100	130

NB. Clinical sensitivity: 90.00% (27/30), (95% CI: 73.47–97.89%)*;* Clinical specificity: 98.00% (98/100), (95% CI: 92.96–99.76%)*;* Positive predictive value: 93.10% (27/29), (95% CI: 77.23–99.15%)*;* Negative predictive value: 97.03% (98/101), (95% CI: 91.56–99.38%)*;* Accuracy: 96.15% (125/130), (95% CI: 91.25–98.74%).

**Table 2:** Clinical sensitivity of the GenBody COVID-19 Ag test (COVAG025) based on Ct values.

Criteria N Gene				
Ct Value	Positive	Negative	Sensitivity	95%CI
≤25	15	0	100%	78.20–100.00%
>25–≤30	11	1	91.67%	61.52–99.79%
>30	0	2	Nd *	Nd *

Nd *: not determined.

**Table 3:** Clinical sensitivity of the GenBody COVID-19 Ag test (COVAG025) based on sample collection date.

Criteria N Gene				
Collection Date	Positive	Negative	Sensitivity	95%CI
0 ≤ 3	7	0	100.00%	59.04–100.00%
≥4	17	2	89.47%	66.86–98.70%
Asymptomatic	3	1	75.00%	19.41–99.37%

**Table 4:** Results of the prospective study using the GenBody COVID-19 Ag test (COVAG025).

Evaluation Results of Test Equipment (GenBody COVID-19 Ag Test (COVAG025)	Confirmed Results through RT-PCR (EURO Real-Time SARS-CoV-2)		Total
	Positive	Negative	
Positive	94	0	94
Negative	6	100	106
Total	100	100	200

NB. Clinical sensitivity: 94.00% (94/100), (95% CI: 87.40–97.77%)*;* Clinical specificity: 100.00% (100/100), (95% CI: 96.38–100.00%)*;* Positive predictive value: 100.00% (94/94), (95% CI: 96.15–100.00%)*;* Negative predictive value: 94.34% (100/106), (95% CI: 88.09–97.89%)*;* Accuracy: 97.00% (194/200), (95% CI: 93.58–98.89%).

**Table 5:** Clinical sensitivity of the GenBody COVID-19 Ag test (COVAG025) based on Ct values.

Ct Value	Positive	Negative	Sensitivity	95%CI
≤25	49	5	90.74%	79.70–96.92%
>25–≤30	44	1	97.78%	88.23–99.94%
>30	1	0	Nd *	Nd *

Nd *: not determined.

**Table 6:** Clinical sensitivity of the GenBody COVID-19 Ag test (COVAG025) based on sample collection date.

Collection Date	Positive	Negative	Sensitivity	95%CI
0 ≤ 3	64	3	95.52%	87.47–99.07%
≥4	30	3	90.91%	75.67–98.08%

## Data Availability

Data supporting reported results may be provided on reasonable request to the corresponding author.
